# Recent Advances in Organocatalyzed Domino C–C Bond-Forming Reactions

**DOI:** 10.3390/molecules23010033

**Published:** 2017-12-23

**Authors:** Cleo S. Evans, Lindsey O. Davis

**Affiliations:** Department of Chemistry and Biochemistry, Berry College, P.O. Box 495016, Mt. Berry, GA 30149, USA; cleo.evans@vikings.berry.edu

**Keywords:** organocatalysis, domino, tandem, Henry, aldol, Mannich

## Abstract

Reactions that form a C–C bond make up a foundational pillar of synthetic organic chemistry. In addition, organocatalysis has emerged as an easy, environmentally-friendly way to promote this type of bond formation. Since around 2000, organocatalysts have been used in a variety of C–C bond-forming reactions including Michael and aldol additions, Mannich-type reactions, and Diels–Alder reactions, to name a few. Many of these methodologies have been refined and further developed to include cascade and domino processes. This review will focus on recent advances in this area with an emphasis on methodologies having applications in the synthesis of biologically-significant compounds.

## 1. Introduction

Reactions that form carbon–carbon bonds are an important tool for a synthetic organic chemist. Since the early 2000s, the field of organocatalysis has developed as an attractive alternative to traditional metal Lewis acid catalysis [1]. As the field of organocatalysis has matured, chemists have found methods for many organocatalyzed domino processes allowing for the synthesis of complex molecules in an efficient manner [2]. The aim of this mini-review is to highlight the work done over the past two years in organocatalyzed domino processes that involve the formation of a carbon–carbon bond. In particular, we will focus on reports that have led to the synthesis of compounds with biological and medicinal significance. Also, we have tried to avoid reviewing literature that has been recently cited elsewhere [3].

## 2. Mannich

The Mannich reaction is an extensively-studied and significant tool in the synthesis of β-amino ketones, sometimes referred to as Mannich bases [4,5]. Organocatalyzed, asymmetric variants of this reaction were developed in the early 2000s, primarily utilizing proline and its derivatives as the organocatalyst [6], although *Cinchona* alkaloids [7,8], (DHQD)_2_-based catalysts (dihydroquinidine) [9], thioureas [10], and phosphoric acid derivatives [11] have been employed as well. Tandem organocatalyzed Mannich reactions have been reported in the synthesis of biologically-significant compounds [12], but many of these reactions have been reviewed elsewhere [13]. The focus of this section will be tandem Mannich reactions reported between 2016 and late 2017.

**Scheme 1:**
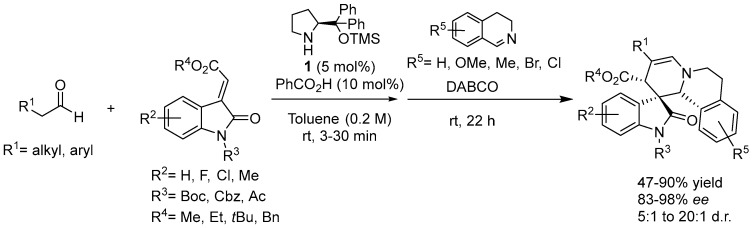
Michael–Mannich–hemiaminalization–dehydration cascade.

Spirocyclic oxindole scaffolds are found in a variety of biological molecules [14,15,16,17,18]. Yu and co-workers recently reported a synthesis of spirooxindole benzoquinolizines utilizing a Michael–Mannich–hemiaminalization–dehydration cascade (Scheme 1) [19]. The Jørgensen–Hayashi catalyst (**1**) was used to catalyze the reaction and 10 mol % PhCO_2_H was used an additive. After screening various bases for the Mannich reaction, DABCO (1,4-diazabicyclo[2.2.2]octane) was chosen as the best base, as compared to DIPEA (*N*,*N*-diisopropylethylamine), K_2_CO_3_, or PPh_3_ among others. The scope of the reaction, made up of 19 examples, included both electron-donating and electron-withdrawing groups at the phenyl ring of the imine, although no strong electron-withdrawing groups were employed. In addition, a gram scale synthesis was achieved with good yield (65% yield), showing promise for this reaction methodology to be used in commercial production.

**Scheme 2:**
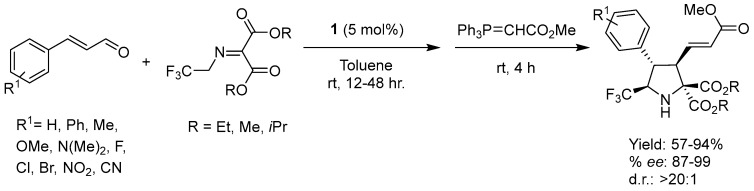
Michael–Mannich cascade followed by Wittig olefination.

Catalyst **1** has also recently been used to catalyze a Michael/Mannich [3 + 2] cycloaddition cascade reaction between α-β-unsaturated aldehydes and trifluoromethyl-substituted iminomalonates to form trifluoromethyl-substituted pyrrolidines (Scheme 2) [20].

Substituted pyrrolidines are found in many bioactive natural products and pharmaceutical agents [21,22]. Particularly, the addition of the trifluoromethyl group to these pyrrolidines makes them attractive targets for synthetic organic chemists [23,24]. This methodology provides a synthesis of chiral trifluoromethylated pyrrolidines with good yield and excellent diastereoselectivity and enantioselectivity. In addition, the reaction proceeded smoothly with electron-withdrawing and electron-donating groups appended to the α,β-unsaturated aldehyde.

**Scheme 3:**
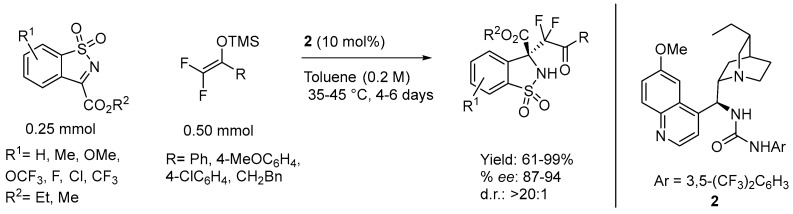
Formation of fluorinated benzosultam derivatives.

In addition to proline-derived organocatalysts, urea-derived organocatalysts have been used in domino reactions. Zhou and co-workers recently developed an asymmetric Mukaiyama–Mannich reaction between fluorinated silyl enol ethers and ketimines catalyzed by a hydroquinine derived bifunctional urea catalyst **2** (Scheme 3) [25].

Benzosultam based C^α^-tetrasubstituted α-amino acid derivatives, the products of this reaction, have been shown as valuable chiral auxiliaries and reagents [26,27,28,29] and found in biologically-significant compounds [30,31]. Through a catalyst screening, the authors determined that both N–H bonds of the urea catalyst were necessary for achieving enantiofacial control. The substrate scope included difluoroenoxysilanes with appended electron-donating and electron-withdrawing groups; however, aliphatic difluorinated enol silyl ethers were not reactive enough to give a substantial amount of product. The reaction was scalable to a 3.0 mmol scale, showing the potential of this methodology for practical synthetic purposes. In addition to difluorinated substrates, monofluorinated enol silyl ethers were also successful substrates in this reaction, producing monofluorinated benzosultam products in high yield (78–99% yield) and good selectivity (>20:1 dr and 90–92% *ee*).

## 3. Henry Reactions

The nitro-aldol reaction, or Henry reaction, has been established as a powerful methodology for the formation of carbon–carbon bonds between nitroalkenes and ketones or aldehydes [32,33,34]. Though the Henry reaction was discovered in 1896, a chiral variant was not developed until the 1990s [35,36]. Asymmetric organocatalysts were employed in both the Henry reaction and aza-Henry reaction in the early 2000s [37,38]. Since then, researchers have sought to expand the reaction scope and reaction conditions, testing new chiral organocatalysts such as squaramide [39], thiourea derivatives [40], proline derivatives [41], and bipyridine derivatives [42]. Like the aldol reaction, Henry reactions are often used in tandem with Michael reactions [40,41,43,44]. The following syntheses involve Michael–Henry or double-Michael–Henry cascades that proceed via different chiral organocatalysts to form new ring systems with multiple stereogenic centers.

**Scheme 4:**
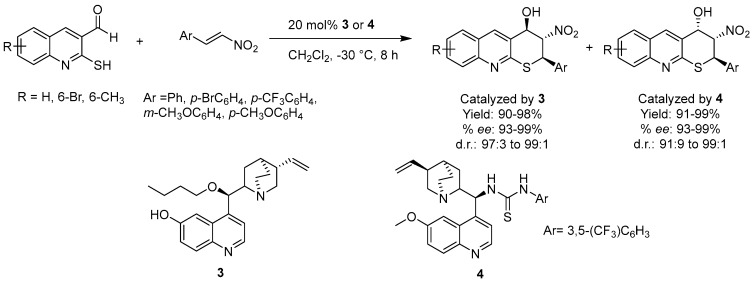
Synthesis of chiral functionalized quinolines via Michael–Henry reaction.

Xie and co-workers reported diastereodivergent syntheses of 2*H*-thiopyrano[2,3*b*]quinolones with three contiguous stereocenters via a domino Michael–Henry reaction (Scheme 4) [45].

Both the quinolone ring and thiopyran functional group have potential as biological and pharmaceutical targets [46,47,48,49,50,51]. Quinoline-derived organocatalysts had previously been identified as successful catalysts for conjugate additions [52,53,54]. Unsurprisingly, the authors could identify two viable catalysts for the tandem Michael–Henry reaction, quinolines **3** and **4**. They discovered that the diastereoselectivity of these catalysts were complementary to each other, where catalyst **3** produced the 1,2-*anti* diastereomer and catalyst **4** gave the 1,2-*syn* diastereomer. Using a starting material previously synthesized by the group, *o*-thiocyanato-(*E*)-cinnamaldehyde [55], nitroolefin, and two different quinoline-derived organocatalysts, the group was able to obtain excellent yields, enantioselectivity, and diastereoselectivity in 8 h at −30 °C using 20 mol % of catalyst and two equivalents of nitroolefin.

**Scheme 5:**
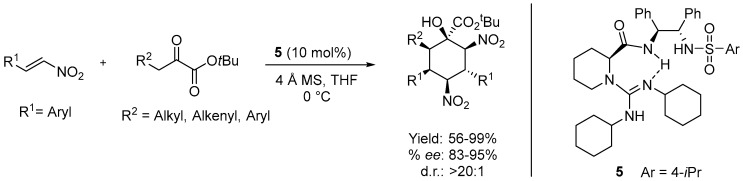
Synthesis of chiral cyclohexane via Michael/Michael/Henry cascade.

Liu and coworkers also used a bifunctional organocatalyst, guanidinium derivative **5**, in a Michael/Michael/Henry cascade (Scheme 5) [56]. Remarkably, this is the first example of a single chiral organocatalyst being employed to create cyclohexanes with six vicinal stereogenic centers. Prior to this publication, a combination of organocatalysts [57] or a Lewis acid catalyst with an organic base co-catalysts [58] were required to achieve this transformation.

The reaction tolerated a variety of electron-donating and electron-withdrawing groups on the aryl substituent of the nitroalkene, although the electronic characteristics played a role in the yield. Those aryl groups with electron-withdrawing substituents typically gave higher yields of the cyclohexane product. The scope of the reaction with respect to the α-ketoester included alkyl, alkenyl, and aryl substituents with little effect on the yield or selectivity. While the utility of the products has not been fully realized, this report provides a remarkable example of an organocatalyzed domino reaction.

**Scheme 6:**
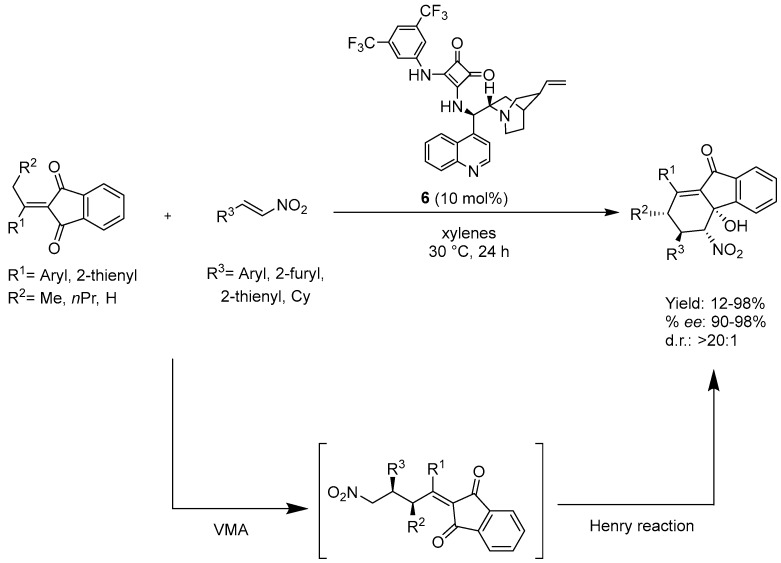
Synthesis of tetrahydrofluoren-9-ones via Michael/Michael/Henry cascade.

Another noteworthy example of an organocatalyzed domino Henry reaction was recently reported by Lin and co-workers. A squaramide derivative **6** was found to catalyze the first tandem vinylogous Michael (VMA)/Henry reaction involving a ketone moiety to synthesize tetrahydrofluoren-9-ones (Scheme 6) [59]. This class of compound was incorporated into medicines used to treat and reduce brain and spinal injuries as early as the 1970s, and is still pharmaceutically relevant [60]. With a combined 23 examples, the scope of this reaction included nitroalkenes with aryl rings with various electron-donating and electron-withdrawing substituents. The 1,3-indandione-derived substrate typically tolerated aryl and alkyl groups.

**Figure 1:**
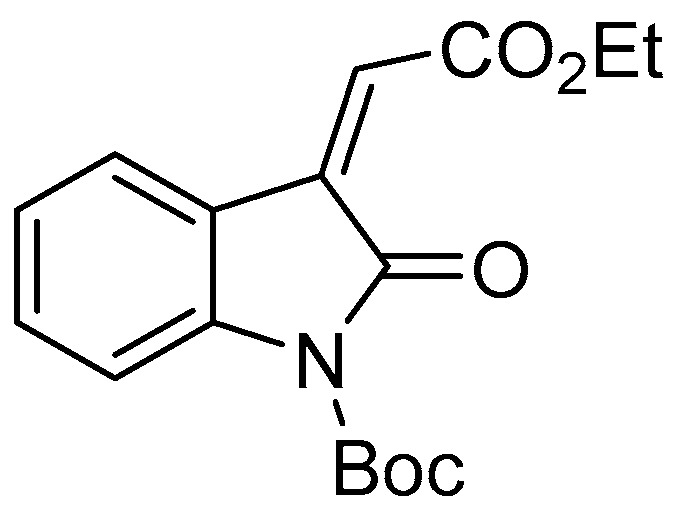
An oxindole derivative used as a Michael acceptor in a VMA/Aldol reaction cascade.

In addition to a wide scope, the authors were also able to provide preliminary results that showed the generality of the indandione-derived pronucleophiles. Another Michael acceptor, a oxindole derivative (Figure 1) was used in this reaction, and with slightly modified reaction conditions, the products were made in high yield (92–98%) and moderate enantioselectivity (81–83%).

**Scheme 7:**
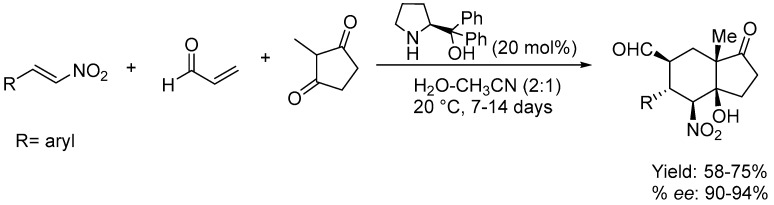
Synthesis of Hajos–Parrish-type ketones via double-Michael/Henry reaction.

Hong et al. constructed highly functionalized Hajos–Parrish-ketones (HPKs) containing five to six contiguous stereogenic centers through an organocatalytic enantioselective Michael/Michael/Henry reaction (Scheme 7) [61]. HPKs have been used as important synthons for a variety of natural products [62], and are one of the historical origins of organocatalysis [63,64]. The Jorgensen–Hayashi catalyst was used in a biphasic system of water:acetonitrile at 2:1 over a seven to fourteen day period at room temperature. The aqueous phase allowed for the necessary dissolution of cyclopentadione reagent. A variety of HPKs were synthesized (10 examples) with moderate to good yield and excellent enantioselectivity.

It is noteworthy that the synthesized HPKs have five to six contiguous stereocenters and two quaternary carbons. This report provides a hallmark example of the utility of domino reactions to create complex products efficiently.

## 4. Aldol Reactions

The aldol reaction is arguably one of the most researched and versatile C–C bond-forming reactions in all of organic chemistry [65,66,67]. Not surprisingly, there are many examples of organocatalyzed aldol reactions, typically catalyzed by proline and its derivatives [68,69,70]. Aldol reactions have commonly been incorporated in domino reactions and reviewed somewhat recently [71,72]; therefore, we will focus on reports from this year only.

**Scheme 8:**
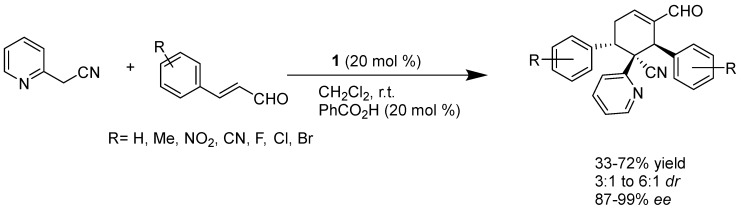
Synthesis of pyridine derivative by Michael/Michael/Aldol reaction.

Rios and co-workers developed a double Michael addition to α,β-unsaturated aldehydes, followed by an intramolecular aldol reaction to synthesize pyridine derivatives using a chiral secondary amine catalyst **1** (Scheme 8) [73].

Remarkably, this cascade resulted in the formation of three C–C bonds with moderate yield and diastereoselectivity and excellent enantiopurity. Enals with appended electron-withdrawing groups (e.g., *p*-nitro, *p*-cyano) were excellent substrates, whereas enal substrates with substituted halogen atoms provided the final products in only moderate yield (63–72%). Unfortunately, if an electron-donating group such as an aliphatic aldehyde was employed as the substrate, the reaction gave a complex mixture. Nevertheless, this report provides an excellent example of the power of the aldol reaction in a domino process.

**Scheme 9:**
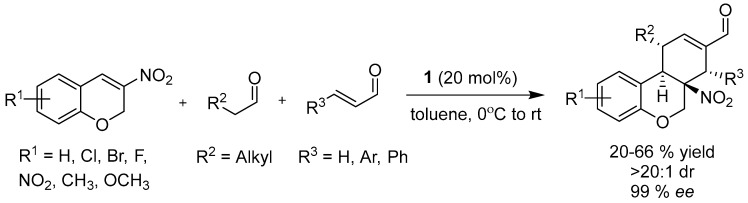
A Michael–Michael–aldol condensation to produce chromanes.

The (*S*)-TMS-diarylprolinol catalyst **1** has also been used recently to catalyze a Michael/Michael/aldol condensation to provide tricyclic chromanes bearing four contiguous stereogenic centers, one of which is tetrasubstituted (Scheme 9) [74]. Chromanes are a commonly-found scaffold in a variety of natural products, some of which have anticancer and antibacterial [75,76], antifungal [77], analgesic [78], and antimalarial properties [79,80]. This methodology has a large reaction scope. Nitrochromenes with appended electron-neutral (H), electron-donating, or electron-withdrawing groups at the C6 or C7 position were excellent substrates in this reaction. In addition, dihalogenated 3-nitro-2*H*-chromenes at the C6 and C8 positions provided products in moderate yield and excellent enantioselectivity. Many aliphatic aldehydes were also used as substrates, with moderate yield of chromene products; however, isovaleraldehyde and *tert*-butyl acetaldehyde were not successful substrates even after five days of reaction. The authors also screened different α,β-unsaturated aldehydes, where aliphatic aldehydes (e.g., acrylaldehyde) provided the desired product in good yield and excellent diastereoselectivity and enantioselectivity. The selectivity remained high for aromatic α,β-unsaturated aldehydes bearing electron-neutral, electron-donating, and electron-withdrawing groups. It is noteworthy that this methodology was shown to be viable on a gram scale, demonstrating the applicability of this protocol.

**Scheme 10:**
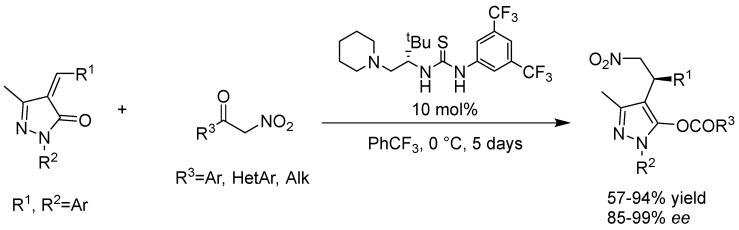
Synthesis of 3-acyloxy pyrazoles through Michael/Hemiketalization/retro-aldol.

Pan and co-workers recently reported a method for the synthesis of 3-acyloxypyrazoles from unsaturated pyrazolones and α-nitroketones through an asymmetric Michael/Hemiketalization/retro-aldol reaction to product 3-acyloxy pyrazoles (Scheme 10) [81].

Pyrazoles are particularly important nitrogen containing motifs because they are found in a wide variety of bioactive compounds [82,83]. A wide variety of pyrazolones having different benzylidene substituents were tolerated in this reaction. Both electron-donating and electron-withdrawing groups at the ortho-, meta-, and para-position of the aryl group afforded the pyrazoles in excellent yields and enantioselectivities. In addition, various pyrazolones with N-substitutions (e.g., 4-MeC_6_H_4_) were also successful substrates with yields of pyrazoles ranging from 81% to 93% with excellent enantioselectivities. The generality of the reaction was further demonstrated as the scope of nitroketones included various those with appended aryl groups, heteroaromatic rings, and alkyl groups.

## 5. Other Reactions

### 5.1. Knoevenegal/Diels-Alder Reactions

**Scheme 11:**
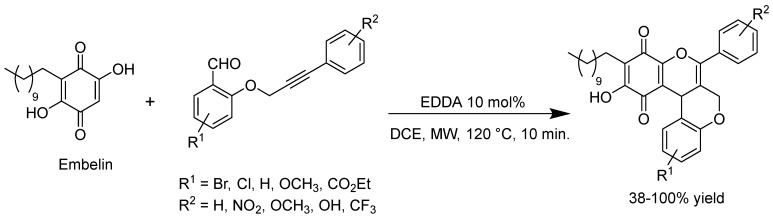
Synthesis of embelin derivatives via organocatalyzed Knoevenegal/Hetero-Diels–Alder domino reactions (DKHDA) reaction.

Estévez-Braun and co-workers have recently reported two examples of Knoevenegal/Hetero-Diels–Alder domino reactions (DKHDA) in the synthesis of embelin derivatives (Scheme 11) [84,85]. Embelin, a biologically-active compound derived from a plant, has been reported to be a promising structural backbone for potential drug candidates [86].

This report is particularly significant because it is the first time intramolecular DKHDA reactions with non-terminal alkynes type *O*-(arylpropynloxy)-salicylaldehydes have been used. Thirty-five aryl-substituted alkynyl ethers were prepared using this methodology, with the majority of reactions giving moderate to high yields of product. The reaction tolerated a variety of electron-donating and electron-withdrawing groups on either aryl ring. The authors hypothesized that the added molecular complexity, introduced with ease because of the domino process, may result in more active and selective biological molecules when compared to embelin.

### 5.2. Wittig Reactions

**Scheme 12:**
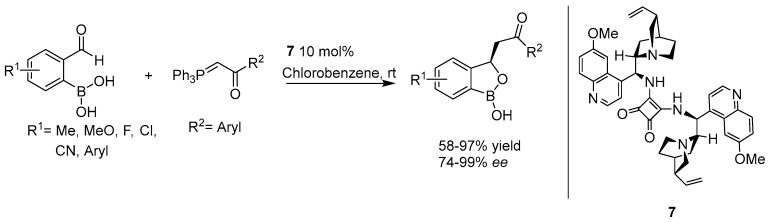
Reaction scope of *o*-formyl aryl boronic acids.

Organocatalyzed Michael/Wittig reactions have been used in the synthesis of pyrazoles [87] and trisubstituted cyclohexene carboxylates [88]. Recently, Ghorai and co-workers reported the use of bifunctional squaramide/thiourea catalyst in a Wittig/oxa-Michael reaction to synthesize benzoxaborole derivatives (Scheme 12) [89].

**Scheme 13:**
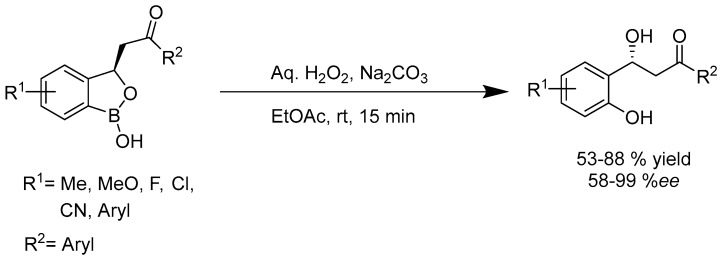
Synthesis of β-hydroxy ketones.

Benzoxaboroles have been shown to have many potential pharmaceutical applications because of their anti-parasitic, antimalarial, anti-inflammatory, antibacterial, and antiviral properties [90]. The bifunctional organocatalyst is thought to coordinate to the carbonyl of the substrates through the squaramide/thiourea functional groups of the catalyst (the pull), and the tertiary nitrogen of the catalyst coordinates to the boron in the substrate providing the “push”. The substitution on the aryl moiety was found to be quite general, as electron-donating and electron-withdrawing substituents worked well, resulting in the isolation of the benzoxaboroles in excellent yield and enantioselectivities. The authors also were able to use the benzoxaborles as substrates in the synthesis of chiral β-hydroxy ketones in good yield and enantioselectivity (Scheme 13).
